# Single-cell and bulk transcriptome analysis unveils a ligand-receptor-based signature for prognostication and reveals that TREM1 controls the malignant behaviors of hepatocellular carcinoma: Ligand-receptor prognostic signature and TREM1-driven HCC malignancy via single-cell/bulk analysis

**DOI:** 10.3724/abbs.2025059

**Published:** 2025-06-23

**Authors:** Jiemin Zhang, Qian Huang, Yingying Zheng, Jianqing Tang, Naling Kang, Yurui Liu, Dawu Zeng

**Affiliations:** 1 Department of Pharmacy the First Affiliated Hospital Fujian Medical University Fuzhou 350005 China; 2 Department of Infectious Disease Union Hospital Fujian Medical University Fuzhou 350000 China; 3 Department of Hepatology Hepatology Research Institute the First Affiliated Hospital Fujian Medical University Fuzhou 350005 China; Clinical Research Center for Hepatopathy and Intestinal Diseases of Fujian Province Fuzhou 350005 China; National Regional Medical Center Binhai Campus of the First Affiliated Hospital Fujian Medical University Fuzhou 350212 China; 4 Department of Pathology the First Affiliated Hospital Fujian Medical University Fuzhou 350005 China

**Keywords:** hepatocellular carcinoma, ecosystem, immune microenvironment, ligand-receptor interaction, TREM1

## Abstract

The transcriptional heterogeneity and cellular ecosystem diversity of HCC await further exploration. Single-cell and bulk RNA sequencing data from HCC cells are analyzed to generate a LASSO model for HCC prognostication. CCK-8, scratch assay, flow cytometry, and ROS assays are used to validate how TREM1 may affect HCC cell biological behaviors *in vitro*. qPCR, western blot analysis, immunohistochemistry, and flow cytometry are applied in a xenograft model to test the effects of *TREM1* knockdown on carcinogenesis and the tumor microenvironment. A single-cell atlas of the multicellular ecosystem comprising 13 cell types in HCC is constructed. On the basis of ligand-receptor marker genes specifically extracted from the cell populations, a prognostic model is defined and subsequently validated in additional clinical cohorts. For the first time, a heterogeneous immune microenvironment is observed between low- and high-risk patients, primarily involving macrophages, CD4+ T cells, M1 macrophages, and regulatory T (Treg) cells. Sufficient evidence validates the positive effects of TREM1 on HCC cell proliferation, migration, and apoptosis. Additionally, TREM1 positively modulates the levels of the proinflammatory cytokines IL-1β, TNF-α, and MCP-1. TREM1 downregulation alters the proportions of M1 macrophages and Tregs in the tumor tissue from our HCC xenograft model. Eventually, the Nrf2/Keap1 signaling pathway, which is related to oxidative stress, is shown to be a key pathway downstream of TREM1 downregulation. In summary, we construct a novel prognostic model for HCC on the basis of ligand-receptor marker genes and investigate the role of TREM1 in HCC progression and its impact on the TME.

## Introduction

Hepatocellular carcinoma (HCC) is a highly aggressive malignancy with a rising incidence globally. The prognosis is still dismal, with an overall five-year survival rate of 12%–15% [1]. In the past few years, remarkable progress has been made in treatment regimens: antiangiogenic multikinase agents (sorafenib [2] and lenvatinib [3]) have gained approval as first-line systemic therapeutic options for unresectable HCCs; other multikinase inhibitors as well as immune checkpoint blockade agents against PD-1/PD-L1 have been approved as second-line treatments [4]. Nevertheless, unresectable HCC patients still face increased unmet medical needs and unsatisfactory survival outcomes. A tumor is an extremely complex ecosystem, defined by spatiotemporal relationships between heterogeneous cell populations composed of malignant, immune, and stromal cell types [5]. Hence, characterizing the landscape of the HCC multicellular ecosystem and critical components linked to tumor progression and immunotherapy is essential.

Using bulk transcriptome approaches, prior research has revealed that each HCC tumor has its own personalized expression profile containing diverse transcriptional programs [6]. Despite the progress in bioinformatics, deconvolution algorithms are unable to analyze rare cell populations and cell-to-cell interplay, among other methods. Single-cell RNA sequencing (scRNA-seq) has been proven to be an efficient tool for characterizing expression data across numerous cells simultaneously, which enables the generation of integrated profiles of diverse cell types in tumors under different biological states or conditions [7]. Recently, scRNA-seq research has provided unique insights into many aspects of HCC biology. For example, scRNA-seq reveals the immunosuppressive landscape and tumor heterogeneity of HBV-related HCC [8]. Single-cell analysis has revealed that proliferative Prom1+ tumor-propagating cells, as well as their dynamic cellular transitions, are involved in HCC development [9]. Through single-cell transcriptomic profiling, the landscape of intratumoral heterogeneity and stemness-associated subpopulations has been revealed in HCC [10].

In the present study, we integrated single-cell and bulk transcriptome analyses to reveal a novel ligand-receptor-based prognostic model for HCC and revealed the role of TREM1 in controlling the malignant behaviors of HCC cells both *in vitro* and *in vivo*. With these insights, our findings revealed that transcriptional heterogeneity and cellular ecosystem diversity are associated with HCC prognosis.

## Materials and Methods

### Single-cell and bulk RNA-seq data acquisition

From the Gene Expression Omnibus, scRNA-seq data from 10 HCC samples were gathered from the GSE149614 dataset ( https://www.ncbi.nlm.nih.gov/geo/query/acc.cgi?acc=GSE149614) [11]. This study also acquired bulk transcriptome data and data on the clinical traits of 365 HCC patients from The Cancer Genome Atlas-Liver Hepatocellular Carcinoma (TCGA-LIHC) ( https://portal.gdc.cancer.gov/repository). Two additional clinical cohorts (GSE14520 and GSE76427) were used to validate the stability of the predictive model in HCC.

### Quality control and preprocessing

The BarcodeRank function of the DropletUtils package was adopted for detecting the expression of single cells, and empty droplets without any gene expression were filtered out via the emptyDrops function [12]. Cells with a number of unique molecular identifiers (UMIs) < 100 were then removed. Through the calculation of the QCMetrics function of the Scater package [13], gene expression in single cells was quantified. Following the criteria of the proportions of mitochondrial genes > 10% and ribosomal genes > 10%, the cells were further filtered out.

### Data normalization

After filtering, the expression matrix was normalized via the normalizeData function of the Seurat package [14]. The first 2000 genes that were highly variable from cell to cell were selected via the FindVariableFeatures function. After linear scaling of the scRNA-seq data with the ScaleData function, principal component analysis (PCA) was carried out on the data for dimensionality reduction analysis with the RunPCA function. Principal components (PCs) with larger standard deviations (SDs) were screened for subsequent analysis.

### Cell clustering, annotation, and marker gene identification

Single cells were clustered using the ‘FindVariableFeatures’ and ‘FindClusters’ functions in the Seurat package. The ‘RunUMAP’ function was implemented to perform dimensionality reduction through uniform manifold approximation and projection (UMAP) [15]. In accordance with the known cell markers from CellMarker 2.0 ( http://biobigdata.hrbmu.edu.cn/CellMarker) [16], the cell clusters were annotated. Marker genes were identified using the ‘FindAllMarkers’ function of the Seurat package, adhering to the criteria of log (fold change) ≥ 0.1, expression proportion in cell clusters > 0.25, and p < 0.05.

### Trajectory analysis

The Monocle tool [17] was employed for trajectory analysis of each cell cluster. Genes expressed in more than 5% of the cells were selected. Using the ‘reduceDimension’ function, dimensionality reduction analysis was implemented, and the cells were clustered via the ‘clusterCells’ function. Afterwards, DEGs (p value < 0.05) between clusters were determined through the ‘differentialGeneTest’ function. On this basis, dimensionality reduction analysis was conducted using the DDRTree method. The cells were then ordered along the trajectory by the ‘orderCells’ function.

### Cell cycle analysis

Following the marker genes of the cell cycle [18], the cell clusters were scored and classified into G1, G2/M and S phases by using the ‘CellCycleScoring’ function in the Seurat package.

### Ligand-receptor network analysis

In accordance with known ligand-receptor relationships [19], known ligand-receptor pairs were downloaded from the DLRP27 ( http://dip.doe-mbi.ucla.edu/dip/dlrp/dlrp.txt), IUPHAR28 ( http://www.guidetopharmacology.org/DATA/interactions.csv) and HPMR29 ( http://receptome.stanford.edu/) databases. After mapping to the current HGNC symbols, we obtained 469, 371 and 855 ligand-receptor pairs from DLRP, IUPHAR and HPMR, respectively. An additional 128 orphan ligands and 479 orphan receptors were also downloaded from HPMR (26 June 2014). The relationship pairs between receptors and ligands in marker genes of cell types were determined. Next, a ligand-receptor network was visualized via Cytoscape software [20].

### Least absolute shrinkage and selection operator (LASSO) analysis

Patients from the TCGA-LIHC dataset were randomized into the discovery set or test set at a 7:3 ratio. On the basis of the marker genes obtained from the ligand-receptor network, as well as bulk transcriptome profiling and clinical information, feature genes were selected from the discovery set using the ‘glmnet’ approach [21]. The risk score was computed using the transcript levels and coefficients of the feature genes. Univariate Cox regression analysis of the feature genes associated with patient survival was conducted. The patients were classified into low- or high-risk subgroups on the basis of the median risk score. Kaplan-Meier curves for overall survival (OS) or disease-free survival (DFS) were plotted using the ‘survival’ package, with the log-rank test used to estimate survival differences. Receiver operating characteristic (ROC) curves were also constructed. The repeatability of the LASSO model was verified in both the test set and the total set. In addition, the risk score was compared across diverse clinical traits.

### Gene set enrichment analysis (GSEA) or single-sample GSEA (ssGSEA)

With the hallmark gene sets obtained from the Molecular Signatures Database as the reference [22], the hallmark enrichment score was computed using GSEA [23] or ssGSEA [24]. The infiltration scores of 28 immune cells were estimated using ssGSEA.

### Cell culture and transfection

HuH-7 and Hep3b HCC cells were purchased from Procell Life Science & Technology Co., Ltd. (Wuhan, China). The cells were cultivated in Dulbecco’s modified Eagle’s medium (DMEM; cat. no. 21969035; Gibco, Grand Island, USA) supplemented with 10% fetal bovine serum (FBS; Gibco) and penicillin-streptomycin (100 μg/mL, P078; Sigma-Aldrich, St Louis, USA) in a humidified environment with 5% CO _2_ at 37°C.

Small interfering RNAs (siRNAs) targeting TREM1 (si-TREM1) and scrambled siRNAs (normal control, NC) were acquired from Sangon Biotech (Shanghai, China). The sequences of the siRNAs that generated efficient knockdown were as follows: si-TREM1-1: 5′-GGAUCAUACUAGAAGACUATT-3′; si-TREM1-2: 5′-GGUCAUUUGUACCCUAGGCTT-3′; and si-Control: 5′-UUCUCCGAACGUGUCACGUTT-3′. The cDNA encoding TREM1 was amplified and subcloned and inserted into the pcDNA3.1 plasmid (Invitrogen, Carlsbad, USA), with the empty plasmid pcDNA3.1 serving as a control. The plasmids were transfected into cells via Lipofectamine 2000 (Invitrogen) in accordance with the manufacturer’s protocols.

### Real-time reverse transcription polymerase chain reaction (qRT-PCR)

Total RNA was extracted with an RNAiso Plus kit (Takara, Dalian, China). cDNA was synthesized via the PrimeScript RT reagent kit (Takara). The primers were shown as follows: *TREM1*, 5′-GAACTCCGAGCTGCAACTAAA-3′ (forward), 5′-TCTAGCGTGTAGTCACATTTCAC-3′ (reverse); *IL-1β*, 5′-ATGATGGCTTATTACAGTGGCAA-3′ (forward), 5′-GTCGGAGATTCGTAGCTGGA-3′ (reverse); *TNF-α*, 5′-CCTCTCTCTAATCAGCCCTCTG-3′ (forward), 5′-GAGGACCTGGGAGTAGATGAG-3′ (reverse); *MCP-1*, 5′-CAGCCAGATGCAATCAATGCC-3′ (forward), 5′-TGGAATCCTGAACCCACTTCT-3′ (reverse); and *GAPDH*, 5′-ACAACTTTGGTATCGTGGAAGG-3′ (forward), 5′-GCCATCACGCCACAGTTTC-3′ (reverse). The transcript levels were detected using the ABI 7500 Fast Real-Time PCR system (Foster City, USA). Relative gene expression was measured using the 2 ^–ΔΔCt^ method.

### Scratch assay

The cells were inoculated in a 6-well plate and grown to confluence. The cell monolayer was scraped with a sterile 10-μL pipette tip, washed twice with PBS, and then cultured in serum-free DMEM. Images of the scratches were recorded at 0 h and 24 h under a light microscope (Nikon, Tokyo, Japan).

### Reactive oxygen species (ROS) measurement

Intracellular ROS were measured using the fluorescent dye 2,7-dichlorofluorescence diacetate (DCFH-DA; Sigma-Aldrich). Fluorescence images were acquired under a confocal laser scanning microscope (Nikon).

### Immunoblotting

The cells were lysed with radioimmunoprecipitation assay buffer plus 1% protease inhibitors for half an hour on ice. The extracted proteins were subjected to sodium dodecyl sulfate-polyacrylamide gel electrophoresis, followed by transfer to a PVDF membrane (Millipore, Billerica, USA). After being blocked, the membrane was incubated with primary antibodies against Nrf2 (1:500; ab137550; Abcam, Cambridge, UK), Keap1 (1:2000; ab227828; Abcam), or GAPDH (1:10,000; ab181603; Abcam), as well as a horseradish peroxidase-conjugated anti-rabbit secondary antibody. Proteins were detected using Pierce™ ECL Western blotting substrate (Sigma-Aldrich ). The images were processed and analyzed using ImageJ software.

### Xenograft assay

BALB/c-Nude mice (6–8 weeks old, female, weighing approximately 20–25 g, SPF grade) were purchased from Changzhou Cavins Laboratory Animal Co. Ltd. [SCXK(SU)2021-0013; Changzhou, China]. HuH-7 cells with stable knockdown of *TREM1* were cultured in DMEM supplemented with 20% FBS, 100 U/mL penicillin, and 100 μg/mL streptomycin in a cell culture incubator with 5% CO _2_ at 37°C. The cells were digested with trypsin when they reached 80%–90% confluence in the culture dish. After centrifugation at 200 *g*, the supernatant was discarded. The cells were washed with PBS and resuspended in PBS, and the cell concentration was adjusted to 2 × 10 ^6^ cells/mL. A xenograft model was then established. Six mice were allocated to each experimental group. Following disinfection with iodophor, a 1-mL syringe was used to aspirate 0.2 mL of the mixed cell suspension (NC/si-TREM1-1/si-TREM1-2) at a density of 2 × 10 ^6^ cells/mL. The needle of the syringe was inserted through the animal’s skin and moved to the injection site to slowly inject the cell suspension. Thirty days after the operation, all the mice were euthanized, and the tumor tissues from each group were excised by autopsy for subsequent experimental tests.

### Establishment of an orthotopic liver cancer mouse model

A total of 25 μL and 1 × 10 ^7^ HuH-7-luc cells were directly injected into the livers of BALB/c-Nude mice through an incision in the skin, and the wounds were sutured promptly, followed by anti-inflammatory treatment. Six mice were assigned to each experimental group.

### Flow cytometry

The cells were collected by centrifugation at 300 *g* for 5 min, and the supernatant was discarded. The cells were resuspended twice with pre-cooled PBS and centrifuged again at 300 *g* for 5 min. Then, 300 μL of binding buffer was added to re-suspend the cells. For Annexin V-FITC labelling, 5 μL of Annexin V-FITC was added and mixed gently, after which the cells were incubated for 15 min at room temperature in the dark. For PI labelling, 10 μL of PI stain was added, the mixture was mixed gently, and the cells were incubated for 10 min at room temperature, ensuring that light exposure was avoided. Antibodies against CD68-PE (566386; BD Biosciences, Franklin Lakes, USA), F4/80-PE (ab105156; Abcam), CD11c-APC (ab210306; Abcam), CD4-FITC (ab269349; Abcam) and Foxp3-PE (ab210231; Abcam) were used to label different cell types. Finally, the cells were analyzed by flow cytometry with a FACScan flow cytometer (BD Biosciences).

### CCK-8 assay

We adhered to the instructions provided in the CCK-8 kit manual (C0038; Beyotime Biotechnology, Shanghai, China) as follows. First, 10 μL of the CCK8 solution was added to each well. The plate was subsequently incubated in the incubator for 1 h. Afterwards, the absorbance at 450 nm was measured using an enzyme-linked immunosorbent assay (ELISA) plate reader (Tecan Group Ltd., Männedorf, Switzerland,).

### Immunohistochemistry

The frozen sections were washed 3 times with PBS, heated to boiling at high heat, and then reduced to low heat for 20 min. The sections were naturally cooled, washed 3 more times with PBS, placed in a 3% H _2_O _2_ solution, and incubated at room temperature for 10 min to block endogenous peroxidase. The sections were subsequently washed 3 times with TBS for 5 min each time, shaken dry, and then blocked with 5% BSA for 20 min. After being washed 3 times with PBS, 50 μL of the diluted anti-Ki-67 antibody (1:100; MCE, Monmouth Junction, USA) was added to each section to cover the tissues, and the sections were incubated at room temperature overnight at 4°C. The sections were then washed 3 times with PBS and incubated in a 3% H _2_O _2_ solution for 10 min. The PBS solution was removed, and 50 μL of secondary antibody from the corresponding species was added to each section and incubated at room temperature for 50 min. After washing with PBS, 50 μL of freshly prepared DAB solution was added to each section, and the sections were rinsed with distilled water. The sections were stained with hematoxylin for 25 s, rinsed with running water for 5 min, and washed with distilled water for 1 min. Finally, the sections were immersed in xylene for a few minutes, air-dried, and sealed with drops of neutral gum before being photographed with a microscope (IX71; Olympus, Tokyo, Japan).

### Transwell assay

The cell migration assay was carried out using 8-μm polycarbonate transwell filters (Corning). Matrigel (BD Biosciences) was thawed overnight at 4°C. On the following day, 150 μL of the Matrigel solution was added to each well of a 24-well plate. Subsequently, the plates were incubated at 37°C for 30 min. After that, the cells were treated with clotrimazole for 24 h. Then, HCC cells were seeded into the upper chamber, and DMEM medium supplemented with 10% FBS was added to the lower chamber. The plate was incubated for another 24 h. After incubation, noninvading cells were carefully wiped off. The invading cells were fixed with paraformaldehyde for 15 min, stained with crystal violet for 15 min, and counted under a microscope at 200× magnification. The number of invading cells in six randomly selected fields was determined.

### Statistical analysis

Data were evaluated using R packages (version 3.5.1) or GraphPad Prism software (version 9.0.1). Comparisons between two groups were performed using Student’s *t* test, the Wilcoxon test, or the chi-square test. Comparisons between ≥ 3 groups were conducted via one- or two-way analysis of variance (ANOVA). Pearson’s or Spearman’s test was used for correlation analysis. *P*  < 0.05 was considered statistically significant.

## Results

### A single-cell atlas of the multicellular ecosystem in HCC

**Figure 1:**
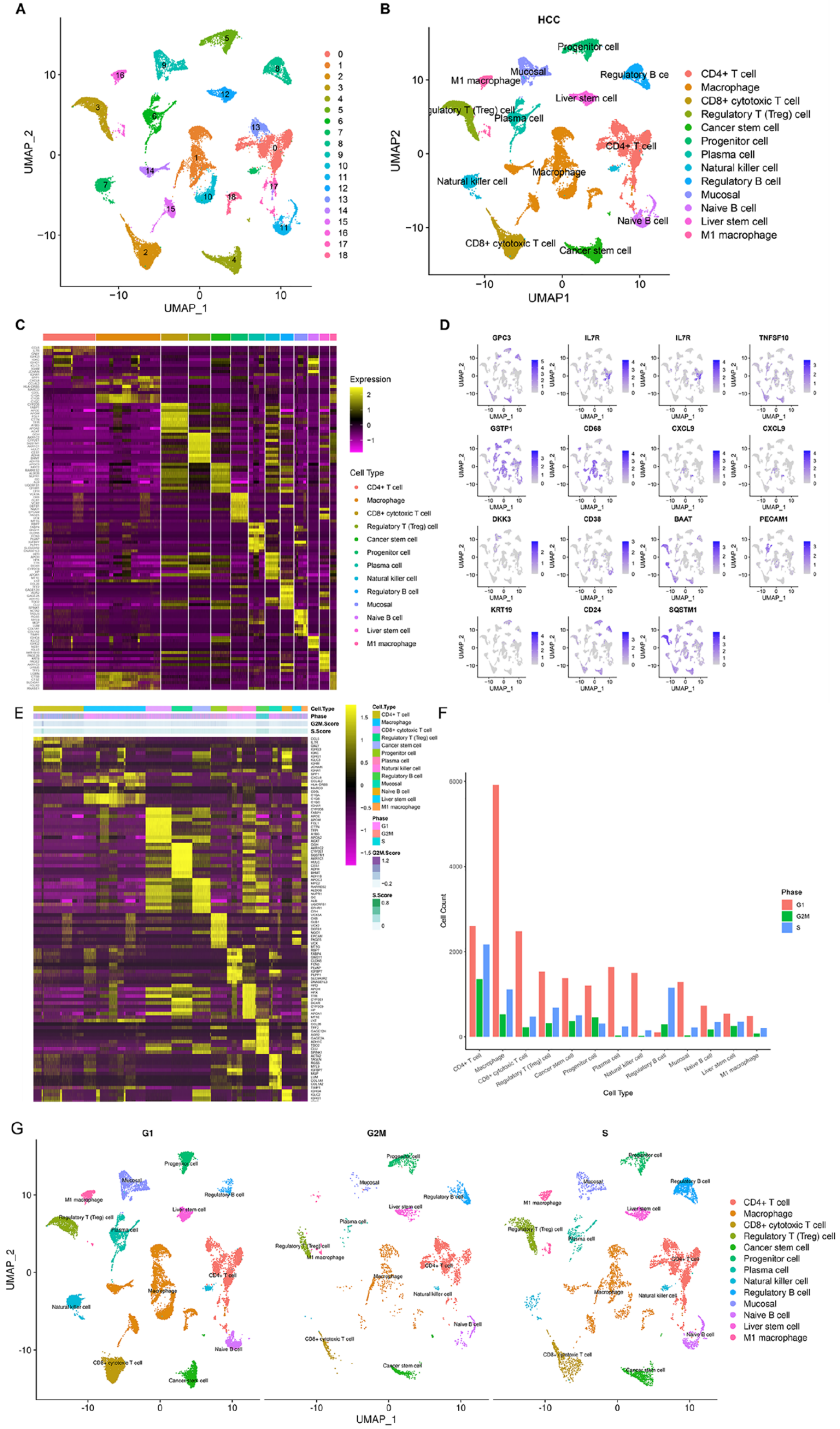
Figure 1 A single-cell atlas of the multicellular ecosystem of HCC (A) UMAP of the cell clusters based on the scRNA-seq data. (B) Cell annotation in accordance with the known marker genes. (C) Heatmap depicting the top 10 marker genes in each cell population. (D) UMAP plot illustrating the top marker gene in each cell population. (E) Heatmap depicting the cell cycle of each cell population. (F) Counts of cells with different cell cycle phases in each cell population. (G) UMAP of the cell clusters annotated with cell cycle information on the basis of the scRNA-seq data.

We initially collected single-cell RNA sequencing (scRNA-seq) data from hepatocellular carcinoma (HCC) for further evaluation. After quality control and preprocessing ( Supplementary Figure S1A–E), as well as data normalization ( Supplementary Figure S2A–E), single cells were clustered into 19 distinct cell clusters ( Figure 1A). Following cell annotation, 13 cell populations were identified, comprising cancer stem cells ( *n* = 2256), liver stem cells ( *n* = 1149), progenitor cells ( *n* = 1976), mucosal cells ( *n* = 1544), CD4 ^+^ T cells ( *n* = 6137), CD8 ^+^ cytotoxic T cells ( *n* = 3187), regulatory T (Treg) cells ( *n*  = 2540), plasma cells ( *n*  = 1910), naive B cells ( *n*  = 1253), regulatory B cells ( *n*  = 1557), natural killer cells ( *n*  = 1678), macrophages ( *n*  = 7555), and M1 macrophages ( *n*  = 779) ( Figure 1B). Figure 1C illustrates the top 10 marker genes of each cell population. Additionally, the top marker for each population is presented ( Figure 1D). Among the cell populations, macrophages and CD4 ^+^ T cells, along with their major subgroups, M1 macrophages and regulatory T (Treg) cells, respectively, were selected as the key cell subgroups for subsequent analysis.

### Cell cycle distribution and trajectory analysis of key cell populations

The cell cycle phase of each cell population was scored on the basis of the expression of marker genes ( Figure 1E). The distribution of cells in the G1, G2/M, and S phases revealed remarkable heterogeneity across each cell population ( Figure 1F). Most of the key cell populations predominantly remain in the G1 phase, followed by the S and G2/M phases. Notably, G1-phase macrophages presented the highest cell count among all the phases ( Figure 1G).

**Figure 2:**
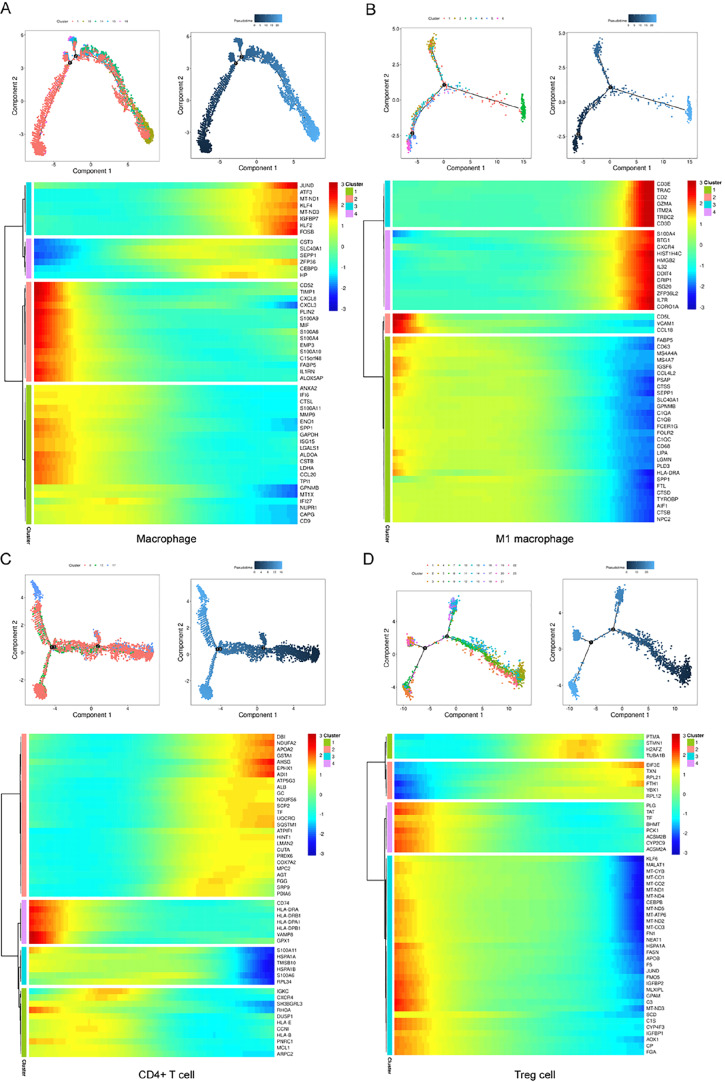
Figure 2 Trajectory analysis of the key cell populations Clustering trajectories and pseudotime trajectories of (A) macrophages, (B) M1 macrophages, (C) CD4+ T cells, and (D) Treg cells. Each dot denotes one cell.

To further investigate cell differentiation, we then determined the pseudotime of various cell populations. Using the Monocle tool, we conducted differential gene testing and branched expression analysis modelling, the results of which are presented in Supplementary Tables S2 and S3. As shown in Figure 2, both macrophages and CD4 ^+^ T cells, as well as their major subgroups, exhibited clearly distinct patterns of pseudotime trajectories and gene expression. The pseudotime trajectories for each cell population are included in Supplementary Figure S3.

### Establishment of a novel HCC prognostic model based on ligand-receptor-mediated multicellular network

**Figure 3:**
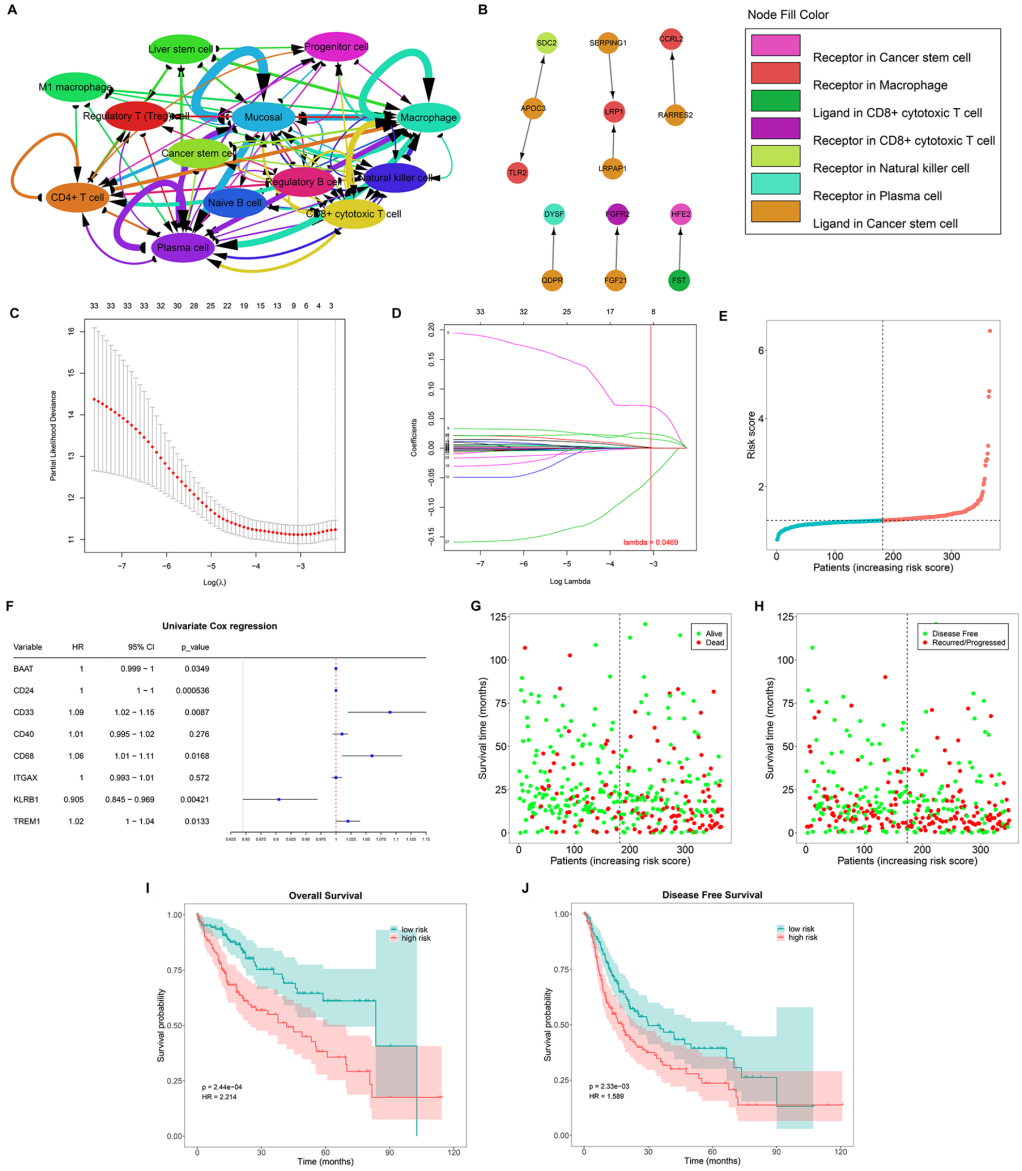
Figure 3 The depiction of a ligand-receptor-mediated multicellular network and generation a ligand-receptor-based prognostic model for HCC (A) Cell-cell interaction network based upon ligand-receptor pairs. (B) The cancer stem cell-based ligand-receptor network. (C,D) Determination of the appropriate lambda value and coefficients of the ligand-receptor genes by LASSO analysis. (E) Distribution of the risk score across the TCGA-LIHC cohort. (F) Univariate Cox regression results for the feature genes associated with HCC survival. (G) Alive or dead status according to the risk score. (H) Disease-free status or recurrence/progression status according to the risk score. (I) OS probability of low- or high-risk patients in the discovery set. (J) DFS probability of low- or high-risk patients in the discovery set.

By taking advantage of the known ligand-receptor relationships involving the marker genes of each cell type, we constructed a ligand-receptor-mediated multicellular network ( Figure 3A). It was found that cancer stem cells occupied the center of the network. Consequently, we identified five cancer stem cell-based interactions with macrophages, CD8 ^+^ cytotoxic T cells, natural killer cells, and plasma cells and matched 33 ligand-receptor marker genes, including *ANPEP*, *BAAT*, *CD14*, *CD163*, *CD24*, *CD33*, *CD40*, *CD63*, *CD68*, *CD69*, *CD81*, *CD86*, *CD9*, *CSF1R*, *CXCL10*, *CXCL2*, *CXCL9*, *FCGR1A*, *FCGR2A*, *FCGR2B*, *FCGR3A*, *GPC3*, *GPNMB*, *IL7R*, *ITGAM*, *ITGAX*, *KLRB1*, *LAMP2*, *LGALS3*, *PECAM1*, *TLR2*, *TNFSF10*, and *TREM1* ( Figure 3B).

We then defined a ligand-receptor-based signature for HCC prognostication. All of the ligand-receptor marker genes were included in the LASSO analysis. We randomized the TCGA-LIHC cohort into discovery and test sets at a ratio of 7:3 ( Supplementary Table S1). With a minimum lambda value of 0.0469, eight feature genes ( *BAAT*, *CD24*, *CD33*, *CD40*, *CD68*, *ITGAX*, *KLRB1*, and *TREM1*) were identified in the discovery set ( Figure 3C,D). The risk score was calculated using the following formula: risk score = (–9.34 × 10 ^–5^) × BAAT + 0.000283442 × CD24 + 0.070615579 × CD33 + 0.000299925 × CD40 + 0.024149562 × CD68 + 0.001050096 × ITGAX + (–0.049868764) × KLRB1 + 0.015595402 × TREM1 ( Figure 3E). Patients were classified into low- or high-risk subgroups on the basis of the median risk score. Among the feature genes, *BAAT*, *CD24*, *CD33*, *CD68*, *KLRB1*, and *TREM1* exhibited notable associations with patient survival ( Figure 3F). More deceased and recurrent/progressive patients were observed in the high-risk subgroup ( Figure 3G,H). Additionally, high-risk individuals had shorter overall survival (OS) and disease-free survival (DFS) durations ( Figure 3I,J), and ROC curves confirmed the favorable predictive performance ( Supplementary Figure S4A,B).

**Figure 4:**
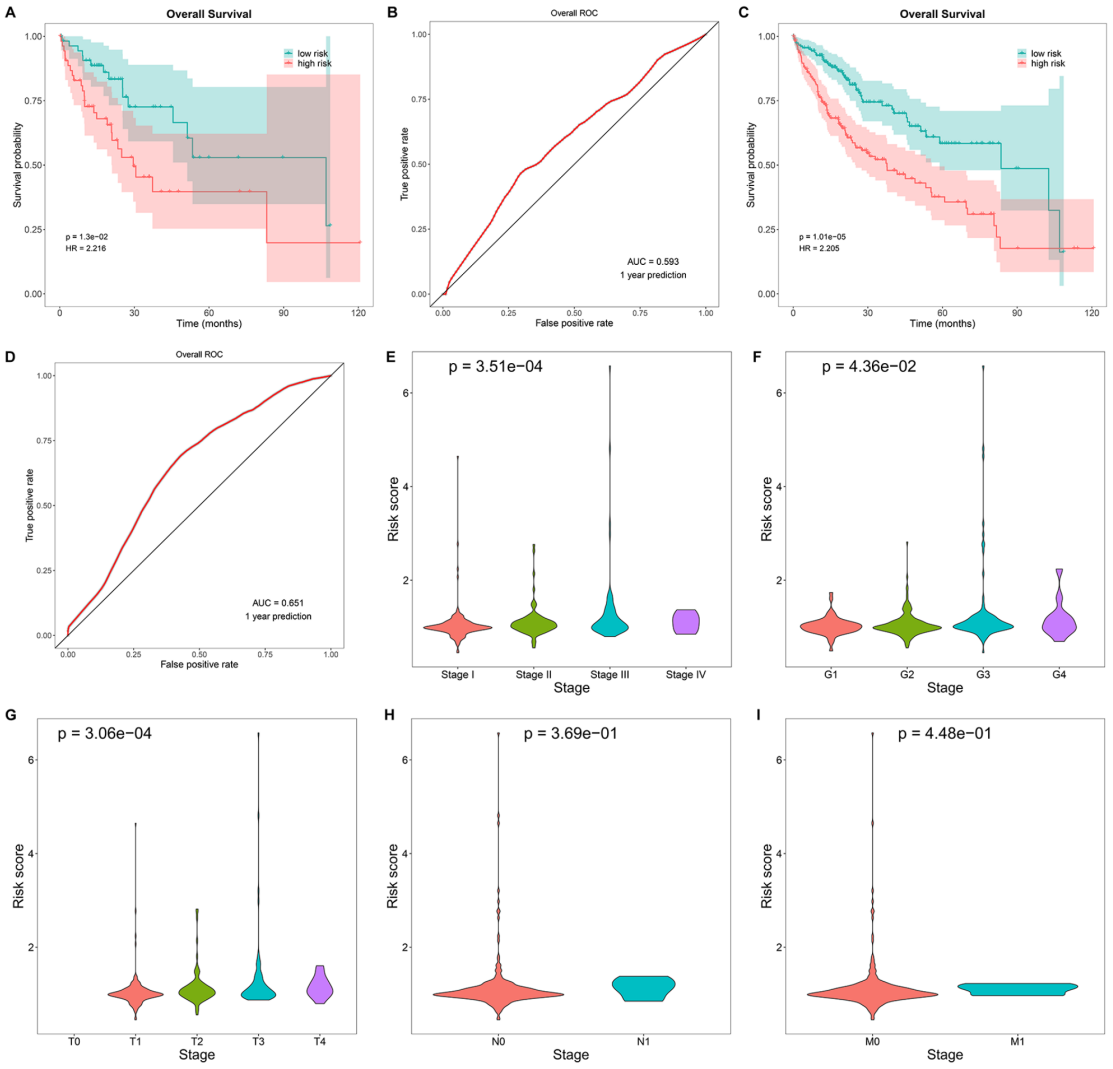
Figure 4 Verification of the repeatability of the risk score and its connections with clinicopathologic traits (A,B) OS probability of low- or high-risk patients in the test set and the total TCGA-LIHC cohort. (C,D) OS probability of low- or high-risk patients in two additional clinical cohorts (GSE14520 and GSE76427). (E–I) Differences in the risk score according to diverse clinicopathologic parameters. Significant differences between different groups were evaluated using one-way ANOVA.

The stability of our prediction model was then validated. As expected, high-risk individuals exhibited poorer OS, with relatively high area under the curve (AUC) values in both the test set and the entire TCGA-LIHC cohort ( Figure 4A,B and Supplementary Figure S4C,D). Two additional clinical cohorts (GSE14520 and GSE76427), which compared tumor and adjacent non-tumor tissues from HCC patients, were also utilized for validation. The significantly higher OS rates and meaningful AUCs confirmed the reliability and repeatability of the LASSO model ( Figure 4C,D and Supplementary Figure S4E,F).

### Functional assessments of the ligand-receptor-based model of HCC

We therefore compared the following clinicopathologic traits between the high-risk and low-risk groups. Pathologic staging determines the extent of cancer in patients following surgery. The cancer grade is very helpful in assessing the speed of cancer growth and the likelihood of cancer spreading. The TNM staging system is also widely used in clinics to measure the size and extent of the primary tumor, the number of nearby lymph nodes that have cancer, and whether the cancer has metastasized. Advanced pathologic stage, grade, and T, N, or M status were associated with a higher risk score ( Figure 4E–I), partly explaining the shorter survival duration for high-risk patients.

**Figure 5:**
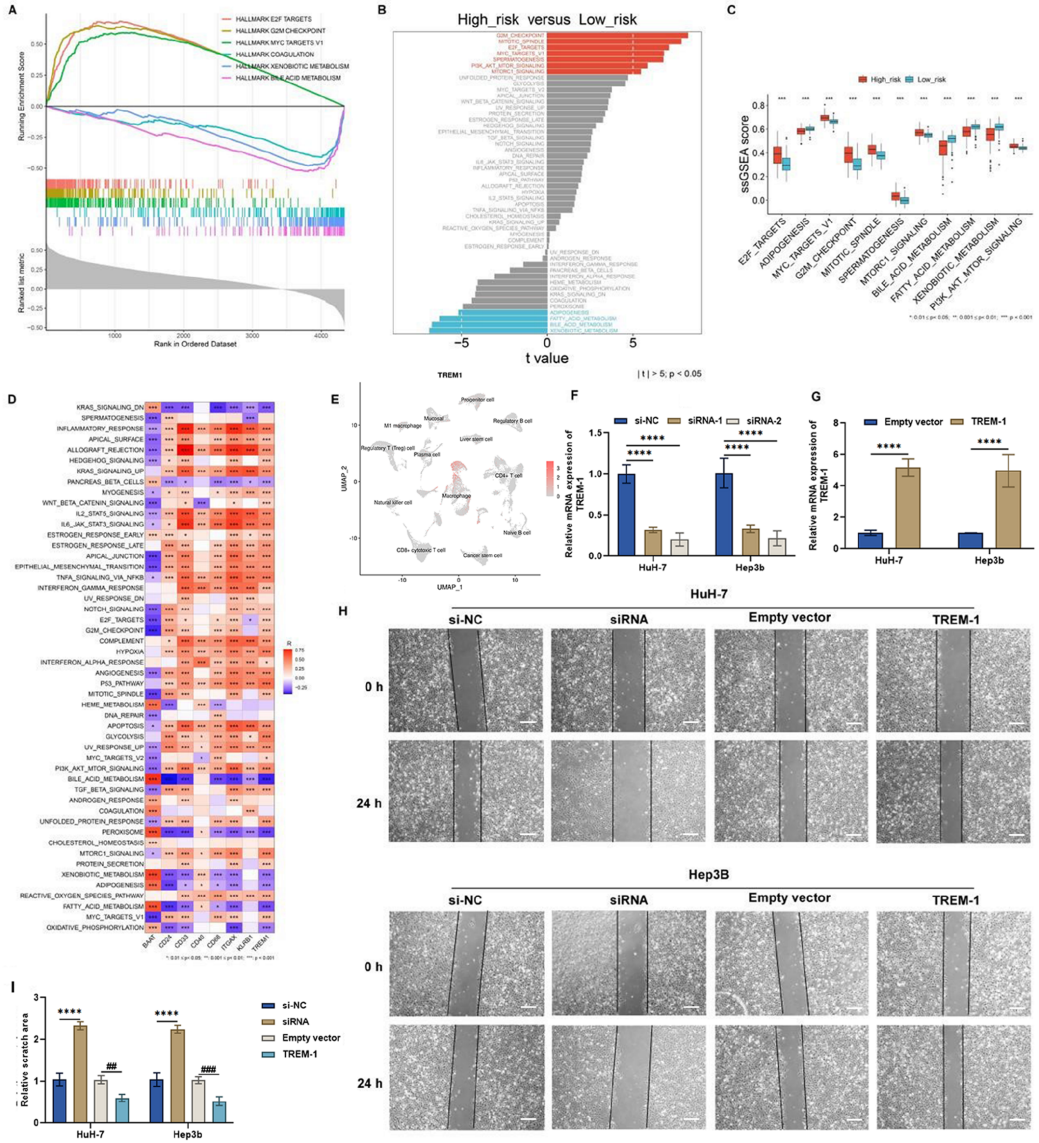
Figure 5 Associations of the risk score with hallmark pathways and the effect of TREM1 on HCC cell migration (A) GSEA of the diversity of hallmark pathways in the low- and high-risk TCGA-LIHC subgroups. (B,C) Comparison of the ssGSEA scores of hallmark pathways between subgroups. (D) Heatmap of the interactions of BAAT, CD24, CD33, CD40, CD68, ITGAX, KLRB1, and TREM1 with hallmark pathways. Blue, negative connection; red, positive connection. (E) TREM1 mRNA levels in HuH-7 and Hep3b cells transfected with siRNAs targeting TREM1. (F) UMAP plot showing the expression of TREM1 in all the cell clusters identified. (G) TREM1 mRNA levels in two HCC cell lines transfected with a TREM1 overexpression plasmid. (H,I) Evaluation of the migration ability of TREM1-silenced or TREM1-overexpressing HCC cells. Statistical results of the invasive ratio after the corresponding treatment for 24 h in the Transwell assay. Scale bar: 20 μm. *P < 0.05, **P < 0.01, ***P < 0.001, ****P < 0.0001 vs si-NC; ##P < 0.01 vs empty vector, ###P < 0.001 vs empty vector.

Next, we identified the differences in functional gene expression between the two risk groups. Gene set enrichment analysis (GSEA) and single-sample GSEA (ssGSEA) demonstrated that E2F targets, the G2M checkpoint, and MYC targets v1 were markedly activated in high-risk samples, with prominent activation of xenobiotic metabolism and bile acid metabolism in low-risk samples ( Figure 5A–C). Additionally, BAAT, CD24, CD33, CD40, CD68, ITGAX, KLRB1, and TREM1 were strongly associated with hallmark pathways ( Figure 5D). Positive interactions included the inflammatory response, IL-2-STAT5 signaling, and TNF-α signaling via NFκB. The negative interactions included oxidative phosphorylation, bile acid metabolism, and others.

**Figure 6:**
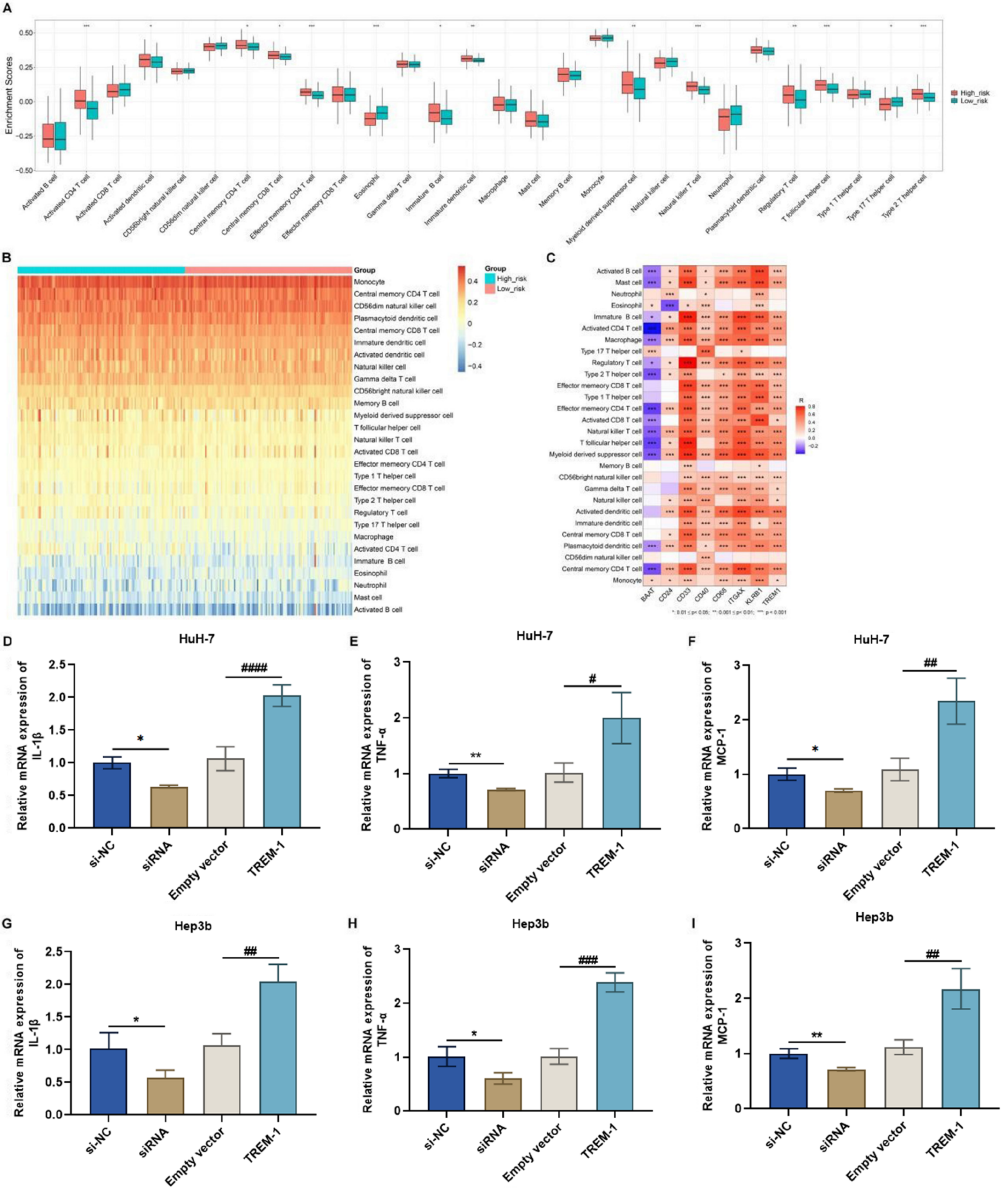
Figure 6 Heterogeneity in the tumor immune microenvironment between low- and high-risk patients and experimental verification of the significance of TREM1 in tumor immunity (A,B) Comparison of the infiltration of diverse immune components in the low- and high-risk TCGA-LIHC subgroups. (C) Heatmap of the connections of BAAT, CD24, CD33, CD40, CD68, ITGAX, KLRB1, and TREM1 with immune infiltration. Blue, negative connection; red, positive connection. (D–F) The levels of IL-1β, TNF-α, and MCP-1 in HuH-7 cells transfected with specific siRNAs or TREM1 overexpression plasmids. (G–I) The levels of IL-1β, TNF-α, and MCP-1 in Hep3b cells transfected with specific siRNA or TREM1 overexpression plasmids. *P < 0.05, **P < 0.01 vs si-NC; ***P < 0.001, ****P < 0.0001 vs empty vector.

We also compared the heterogeneous immune microenvironments between low- and high-risk patients. Activated CD4 ^+^ T cells, immature and activated dendritic cells, central memory CD4 ^+^ and CD8 ^+^ T cells, effector memory CD8 ^+^ T cells, immature B cells, myeloid-derived suppressor cells, natural killer T cells, regulatory T cells, T follicular helper cells, and type 2 T helper cells displayed increased activity in the high-risk subgroup, with decreased activity of eosinophils and type 17 T helper cells ( Figure 6A,B), revealing the heterogeneous immune microenvironment between subgroups. In addition, BAAT was negatively associated with most immune cell types, whereas CD24, CD33, CD40, CD68, ITGAX, KLRB1, and TREM1 exhibited positive interactions with most of the immune components mentioned above. Compared with that of other genes, the role of *TREM1* in the progression of HCC remains less explored. Hence, we further selected TREM1 to investigate its role in HCC both *in vitro* and *in vivo*.

**Figure 7:**
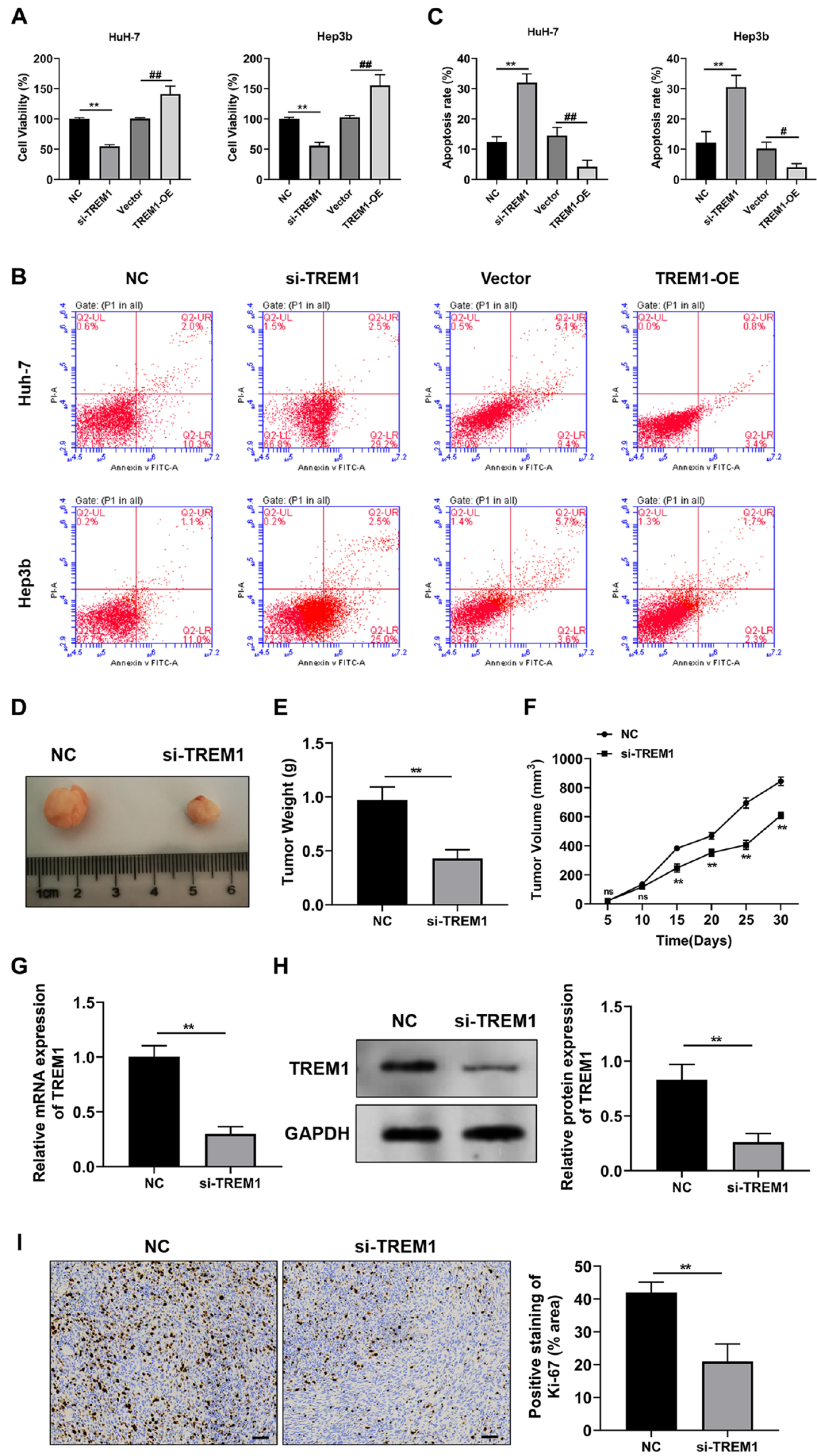
Figure 7 The carcinogenic effects of TREM1 *in vitro* and *in vivo* (A) Viability of Hub-7 and Hep3b cells with TREM1 knockdown or overexpression. (B) Flow cytometry showing the rate of apoptosis in TREM1-silenced or TREM1-overexpressing HCC cells. (C) Histogram summarizing the apoptosis rate under the circumstances mentioned in (B). (D) Representative tumor tissues isolated from xenograft mice injected with HuH-7 cells stably transfected with normal control (NC) or TREM1 siRNA (si-TREM1) are shown. (E,F) The tumor weights (E) and volumes (F) are summarized. (G,H) Relative mRNA expression (G) and protein expression (H) of TREM1 in the tumor tissues of the mice described in (D) onday 30. (I) Representative immunohistochemistry images of Ki-67 expression under the same circumstances as in (D,E). A histogram summarizing the positive staining of Ki-67 is also presented. Six mice were included in each experimental group. **P < 0.01 vs NC; #P < 0.05, ##P < 0.01 vs vector.

### TREM1 controls malignant behaviors and affects the TME of HCC

The biological significance of TREM1 was further experimentally verified. TREM1 expression was notably downregulated by specific siRNAs or upregulated by their overexpression plasmids in HuH-7 and Hep3b cells ( Figure 5E,F). The scratch assay results demonstrated the impaired migration capacity of *TREM1*-silenced HCC cells ( Figure 5G–I). Conversely, TREM1 upregulation increased migration. These data indicate that TREM1 might be responsible for HCC cell migration. The proliferation ability was then analyzed using the CCK8 assay. As shown in Figure 7A, the viability of HuH-7 and Hep3b cells transfected with TREM1 siRNA was significantly lower than that of the NC (negative control) group. Conversely, the viability of Huh-7 and Hep3b cells transfected with the TREM1 overexpression plasmid was significantly greater than that of the empty vector group. The impact of TREM1 on cell apoptosis was also tested. Flow cytometry revealed that *TREM1* silencing clearly increased the rate of apoptosis in HuH-7 and Hep3b cells, whereas TREM1 overexpression had the opposite effect ( Figure 7B,C).

We then explored the anticarcinogenic effects of TREM1 downregulation in a xenograft model. HuH-7 cells stably transfected with NC or si-TREM1 were subcutaneously injected into BALB/c-Nude mice on the left or right side of the body. Both the tumor weight and volume were significantly decreased in the si-TREM1 group ( Figure 7D–F). qPCR and western blot analysis results revealed that, compared with those in the NC group, both the mRNA and protein expression levels of TREM1 were significantly lower in the tumor tissues of mice in which *TREM1* was stably knocked down ( Figure 7G,H). The results of immunohistochemistry also revealed a significant reduction in Ki-67 expression in the tumor tissues of *TREM1*-knockdown mice compared with those of the NC group, indicating that the knockdown of *TREM1* can drastically reduce the proliferation of hepatocellular carcinoma cells ( Figure 7I).

**Figure 8:**
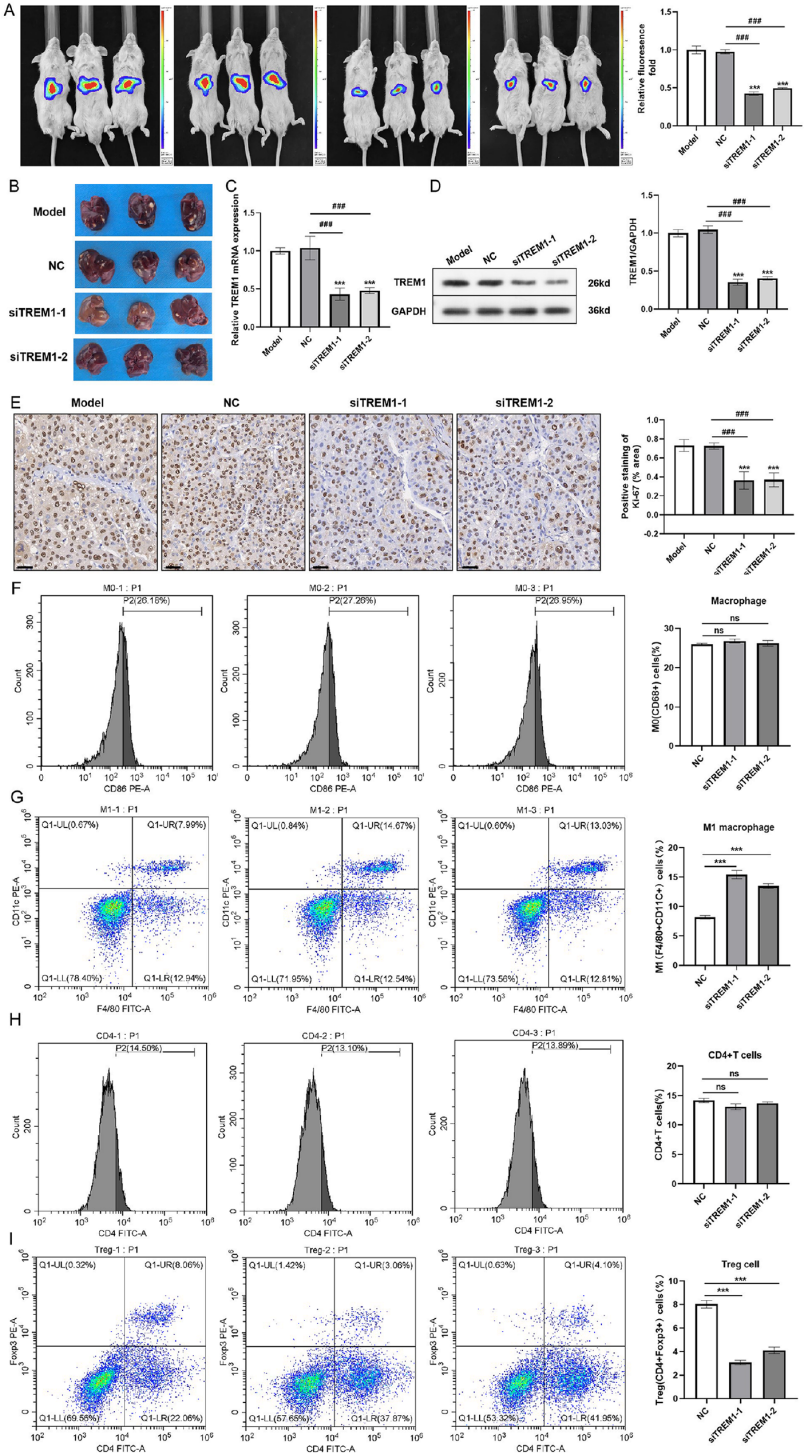
Figure 8 TREM1 controls malignant behaviors and affects the TME of HCC (A) Representative bioluminescent photographs of orthotopic liver cancer-bearing mice in different groups on day 20 after different treatments. The bioluminescence intensity reflects the tumor volume. (B) Digital photographs of liver tissues. (C,D) Relative mRNA expression (C) and protein expression (D) of TREM1 in the tumor tissues of the mice described in (A). (E) Representative immunohistochemistry images of Ki-67 expression under the same conditions described in (A,B). A histogram summarizing the positive staining of Ki-67 is also presented. (F–I) Flow cytometry showing the proportions of M0 macrophages (F), M1 macrophages (G), CD4+ T cells (H) and Treg cells (I) in the tumor tissues of mice in which TREM1 was stably knocked down (si-TREM1-1/si-TREM1-2) or not (NC). ***P < 0.001 vs model; ###P < 0.001 vs NC.

We further investigated whether the TME could be affected by TREM1, as shown in the bioinformatics results ( Figure 6A–C). Inflammation has long been proven to affect the immune microenvironment in tumorigenesis. In terms of inflammatory responses, the levels of the proinflammatory cytokines IL-1β, TNF-α, and MCP-1 were markedly decreased by the suppression of TREM1 in HuH-7 and Hep3b cells ( Figure 6D–I). Conversely, in TREM1-overexpressing cells, the levels of these proinflammatory cytokines were elevated. To assess the impacts on the immune microenvironment, we selectively measured the changes in key cell populations in the tumor tissue from our xenograft model via flow cytometry. Compared with those in the NC group, the proportions of M0 macrophages and CD4 ^+^ T cells in tumor tissues changed slightly after the knockdown of *TREM1*, while the proportion of M1 macrophages increased significantly ( Figure 8A–F). Instead, the proportion of Treg cells in tumor tissues in which TREM1 was downregulated was significantly lower ( Figure 8G–I). This finding was not completely consistent with the results of the bioinformatics analysis ( Figure 6C). Considering the complexity of the TME, more sophisticated experiments and advanced techniques are needed to elucidate how the changes in inflammatory responses caused by TREM1 are responsible for alterations in the immune microenvironment.

**Figure 9:**
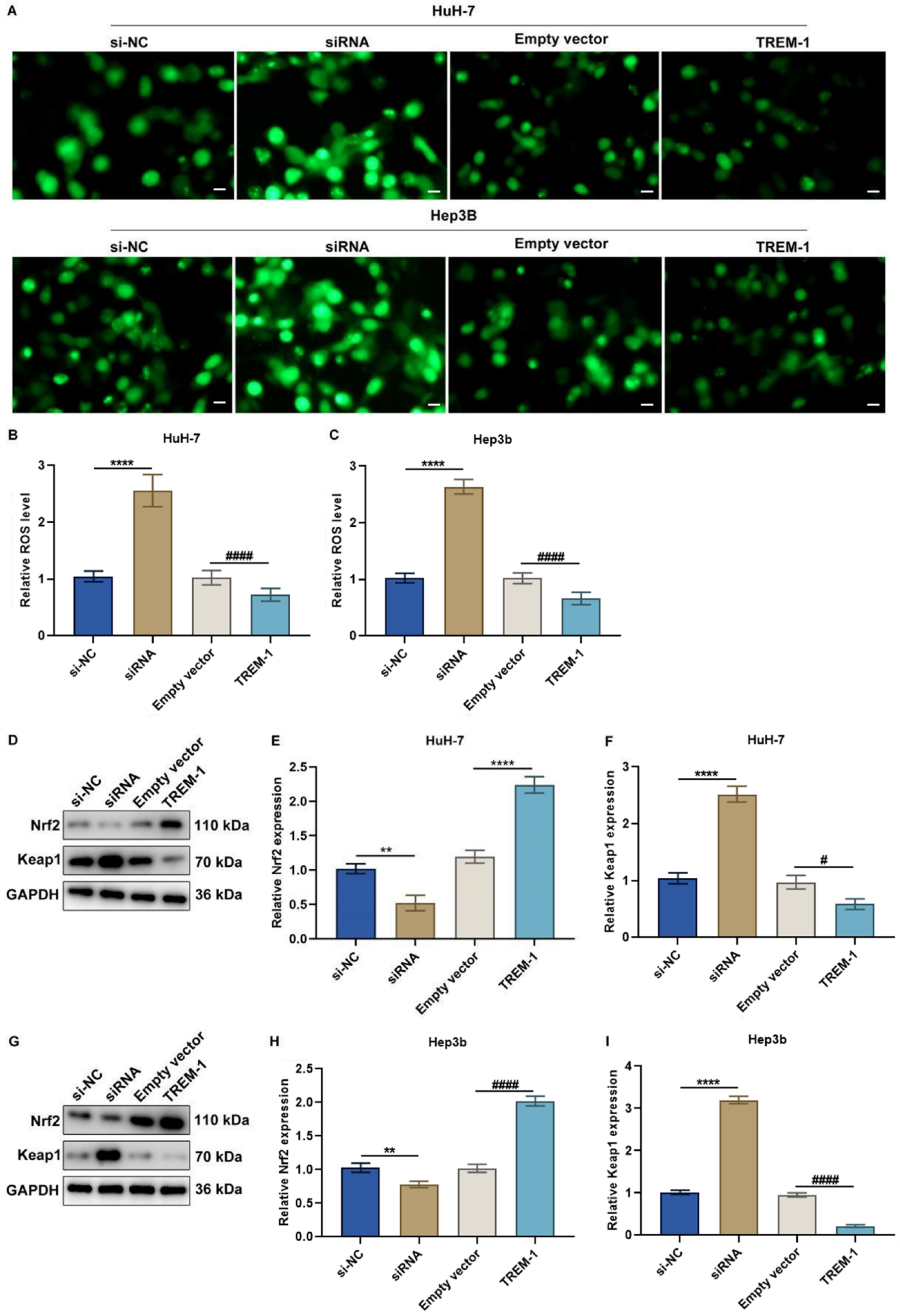
Figure 9 TREM1 protects HCC cells against oxidative stress (A–C) The production of intracellular ROS in HuH-7 and Hep3b cells transfected with specific siRNAs or TREM1 overexpression plasmids. Scale bar: 20 μm. (D–F) Nrf2 and Keap1 expressions in HuH-7 cells transfected with a specific siRNA or TREM1 overexpression plasmid. (G–I) Nrf2 and Keap1 expressions in Hep3b cells transfected with specific siRNAs or TREM1 overexpression plasmids. *P < 0.05, **P < 0.01, ****P < 0.0001 vs si-NC; #P < 0.05, ####P < 0.0001 vs empty vector.

Finally, we explored the key downstream pathways affected by *TREM1* knockdown. Most previous efforts have focused on inhibiting the NF-κB signaling pathway, which mitigates inflammatory responses [ 25, 26]. However, the pathways associated with oxidative stress have been much less investigated in HCC, not to mention their correlation with TREM1. Therefore, we measured the intracellular ROS levels via DCFH-DA. Consequently, significantly increased ROS generation was observed in *TREM1*-silenced HuH-7 and Hep3b cells (siRNA group) compared with that in the NC group ( *P*  < 0.0001; Figure 9A–C). In contrast, compared with empty vector treatment, TREM1 overexpression attenuated the production of intracellular ROS ( *P*  < 0.0001; Figure 9A–C). Nrf2 and Keap1 act as key mediators of oxidative stress [27]. Nrf2 activity was significantly lower, whereas Keap1 activity was clearly greater in *TREM1*-knockdown HuH-7 and Hep3b cells than in control cells (** *P*  < 0.01 and **** *P*  < 0.0001, siRNA group vs si-NC group; Figure 9D–I). The opposite results for Nrf2 and Keap1 activity were observed in TREM1-overexpressing cells ( *P*  < 0.05 and *P*  < 0.0001, TREM1 group vs empty vector group; Figure 9D–I). Taken together, these findings indicate that TREM1 has a protective role in HCC cells against oxidative stress.

## Discussion

In this study, we combined single-cell and bulk transcriptome analysis to establish a ligand-receptor-based signature for HCC prognostication and validated the impact of TREM1 on HCC progression. A single-cell landscape of the multicellular ecosystem in HCC, comprising cancer stem cells, liver stem cells, progenitor cells, mucosal cells, CD4 ^+^ T cells, CD8 ^+^ cytotoxic T cells, Treg cells, plasma cells, naive B cells, regulatory B cells, natural killer cells, macrophages, and M1 macrophages, was first described, thus revealing the diversity of the cellular ecosystem in HCC.

These cell populations closely interact via ligand-receptor pairs. This work defined a ligand-receptor-based signature for HCC prognostication composed of BAAT, CD24, CD33, CD40, CD68, ITGAX, KLRB1, and TREM1. HCC often presents high levels of histological, transcriptomic, and other variations among individual patients, leading to clinical behaviors and therapeutic responses. For the prolongation of HCC patient survival, it is highly necessary to consider such heterogeneity in clinical management as well as to discover the molecular pathways that determine the differentiation of major HCC variations. In this study, cell proliferation pathways (E2F targets, the G2M checkpoint, and MYC targets v1) were found to exhibit notable activation in high-risk individuals, indicating increased proliferation levels in this population. The liver is an extremely dynamic metabolic organ that plays a critical role in bile acid synthesis, xenobiotic metabolism, *etc*. [28]. Remarkable activation of xenobiotic metabolism and bile acid metabolism was observed in low-risk individuals.

In addition to TREM1, there is ample evidence that other genes with a ligand-receptor-based signature for HCC prognostication participate in the development of HCC. For example, BAAT, which mediates primary bile acid synthesis and bile acid conjugation, has been proven to be overexpressed in glutamine synthetase-positive Tsumura-Suzuki obese diabetic-derived HCC tumors [29]. CD24 has been shown to be involved in multiple aspects of HCC [30]. On the one hand, CD24 may result in sorafenib resistance through the activation of autophagy in HCC [31]. On the other hand, CD24 upregulation is correlated with undesirable survival after surgical resection [32] or adjuvant trans-arterial chemoembolization treatment [33]. Moreover, CD33 is associated with an increased risk of HCC among chronic hepatitis B-infected individuals [34]. Myeloid-derived suppressor cells are important for immunosuppression, and their surrogate biomarker, CD33, is related to aggressive tumor phenotypes and short survival durations [35]. Studies have also shown that activated CD40 can improve the immunomodulatory ability of dendritic cells toward gastrointestinal tumors [36]. CD40 ligand-overexpressing dendritic cells are capable of inducing HCC suppression via the activation of innate and acquired immunity [37]. Another signature, CD38, has also been proven to participate in immunosuppressive adenosinergic signaling, and an increased proportion of CD38 ^+^ cells within the immune microenvironment is predictive of the anti-PD-1/PD-L1 therapeutic response in HCC [38]. Furthermore, crosstalk between CD68 and GAS6 in fibroblasts may trigger the recruitment and polarization of macrophages in HCC [39]. Compared with those in primary tumors, CD8 ^+^ T cells in recurrent HCC tumors appear to overexpress KLRB1 and exhibit an innate-like low cytotoxic status [40].

The present work also provides rich evidence for how TREM1 affects HCC cells. Bioinformatics analysis revealed that high expression of TREM1 is related to poor prognosis in patients with HCC [41]. Previous research has reported that pharmacological inhibition or silencing of *TREM1* restrains HCC cell metastasis [42] and that silencing of *TREM1* in macrophages can mitigate the migratory capacity of HCC cells [ 43, 44]. We further observed that TREM1 actively modulated HCC migration and proliferation, accompanied by a decreased level of apoptosis. Similar to previous studies, TREM1 upregulated the proinflammatory cytokines IL-1β, TNF-α, and MCP-1 and resulted in an inflammatory response in HCC cells [45]. TREM1 upregulated the proinflammatory cytokines IL-1β, TNF-α, and MCP-1 and resulted in an inflammatory response in HCC cells. There also appear to be different kinds of possible downstream pathways of TREM1, including the positively interacting inflammatory response, IL-2-STAT5 signaling, and TNF-α signaling via NF-κB, and negatively interacting pathways such as oxidative phosphorylation and bile acid metabolism. Studies have shown that *TREM1* knockdown inhibits the NF-κB signaling pathway to attenuate inflammatory responses [ 25, 26]. We focused on the less investigated pathways related to oxidative stress. We discovered that upregulation of TREM1 helps HCC cells fight against oxidative stress by lowering intracellular ROS generation and mediating Nrf2/Keap1 signaling. Therefore, HCC cells with high levels of TREM1 may continue to thrive and become a serious threat to neighboring healthy tissues.

Currently, increasing attention is being given to changes in the tumor microenvironment (TME) for cancer treatment. The TME comprises mainly tumor cells, their surrounding immune and inflammatory cells, and other cellular and non-cellular components. Immune infiltration is essential for the heterogeneity of the TME, which may lead to the formation of well-known tumor-infiltrating lymphocytes (TILs) that play a critical role in antitumor immunity [46]. However, long-term inflammation can also give rise to tertiary lymphoid structures (TLSs), a double-edged sword in tumorigenesis and metastasis [47]. Therefore, unravelling how these components, especially the immune microenvironment, may react in cancers, including HCC, is highly important. Previous research has reported the enrichment of central memory T cells (TCMs) in early tertiary lymphoid structures (E-TLSs) in HCC and assessed the relationships between chronic HBV/HCV infection and T-cell infiltration and exhaustion [11]. In addition to bioinformatics analysis of the heterogeneity of the immune microenvironment between patients in two risk groups, our present work also investigated the impact of TREM1 on key cell populations in the tumor tissue of a xenograft model. Traditionally, a significant increase in the proportion of M1 macrophages may represent increased proinflammatory responses [48]. However, we cannot exclude the possibility that the M2 type would also increase, thus breaking the M1/M2 balance to induce anti-inflammatory responses as a result. Moreover, the number of Treg cells dramatically decreased in tumor tissue with *TREM1* knockdown. According to previous studies [49], this may thus enhance antitumor immune responses. Considering the complexity and dynamicity of the immune microenvironment, many more explorations are worth performing in the future, from the bench to the bedside.

Despite the breadth of our findings, there are certain limitations in this work. For example, there are currently many models for HCC with much higher AUC values. However, few studies have combined single-cell and bulk RNA sequencing data. Additionally, the marker genes in those studies appear to share more narrowly defined functions, such as autophagy [50], cuproptosis [51] and T-cell depletion [52]. Our present research screened out marker genes by establishing a rather general network, although the AUC values were relatively unsatisfactory. In addition, in-depth experiments are still needed to further prove the importance of TREM1 in HCC pathogenesis. Finally, real-world validation of the prognostic value of TREM1 expression is relatively lacking, although a previous study revealed that high TREM1 expression is significantly correlated with increased recurrence and poorer survival in HCC patients [45]. We also plan to test both the prognostic and treatment value of TREM1 in our clinical cohorts in the future.

Overall, this work depicted the single-cell landscape of the HCC ecosystem and proposed a ligand-receptor-based signature for HCC prognostication on the basis of single-cell and bulk expression analysis. Through experimental verification, we demonstrated that TREM1 may play a crucial role in controlling the malignant behaviors of HCC cells and may be a potential therapeutic target.

## Supporting information

24895Supplementary_table_2

24895Supplementary_Figures

24895Supplementary_Table_1

24895Supplementary_table_3

## Supplementary Data

Supplementary data is available at *Acta Biochimica et Biophysica Sinica* online.
