# Supplementary material for: Single-cell and bulk transcriptome analysis unveils a ligand-receptor-based signature for prognostication and reveals that TREM1 controls the malignant behaviors of hepatocellular carcinoma: Ligand-receptor prognostic signature and TREM1-driven HCC malignancy via single-cell/bulk analysis
Source: Acta Biochim Biophys Sin (Shanghai). 2025 Jun 23;57(11):1847–63. doi: 10.3724/abbs.2025059 (PMC12666666; doi:10.3724/abbs.2025059)
Supplement: 24895Supplementary_Figures [file 24895Supplementary_Figures.docx]

**
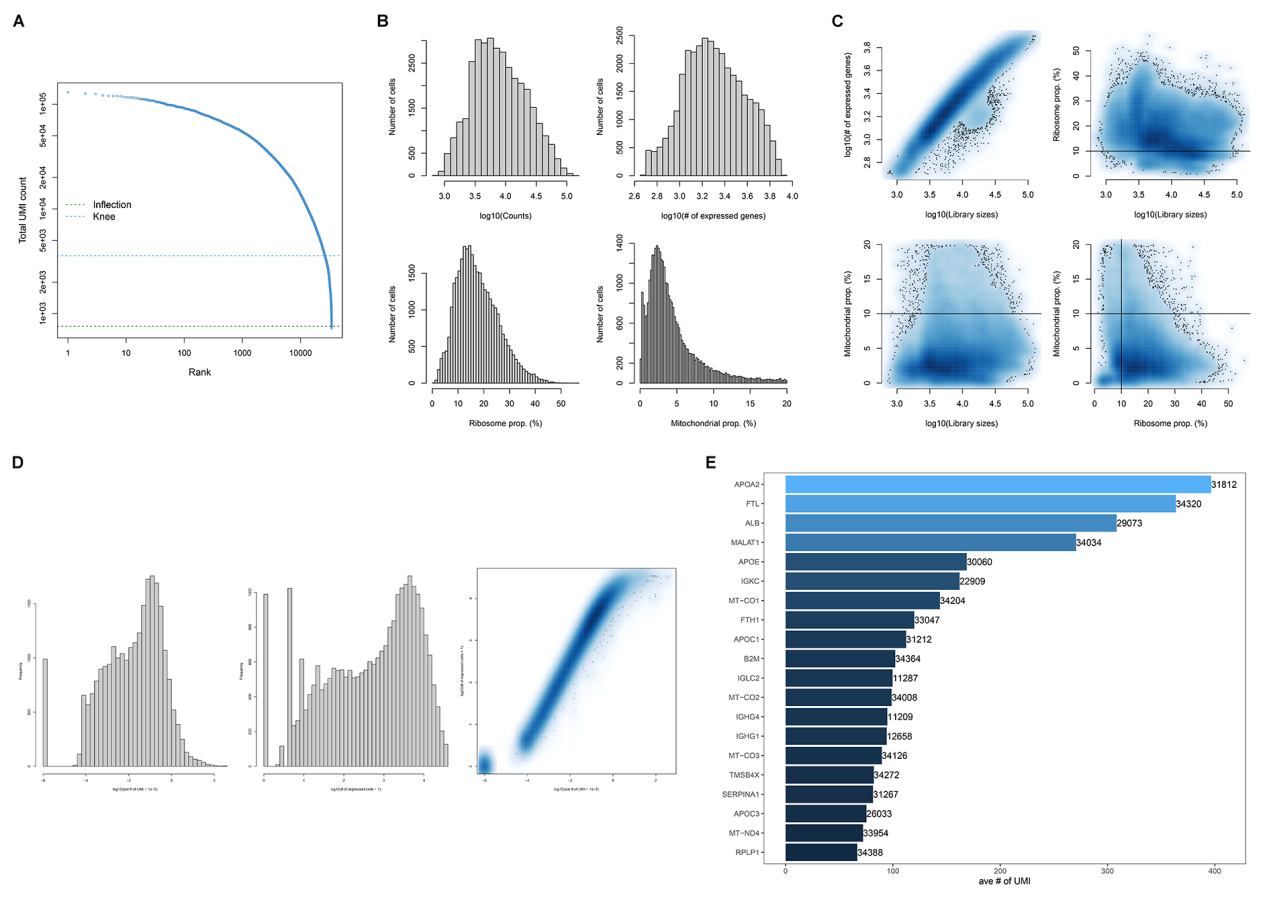
**

**Supplementary Figure S1. Quality control and preprocessing of scRNA-seq data** (A) Total UMI count in single cells following the filtering out of empty droplets without any gene expression. (B) The number of counts and the proportions of expressed genes, ribosomes and mitochondria. (C) Ribosome and mitochondrial proportions following the removal of cells with < 100 UMIs. (D) Expression of single cells following the removal of cells whose proportion of mitochondrial genes was > 10% and whose percentage of ribosomal genes was > 10%. (E) The top 20 genes expressed in most cells.

**
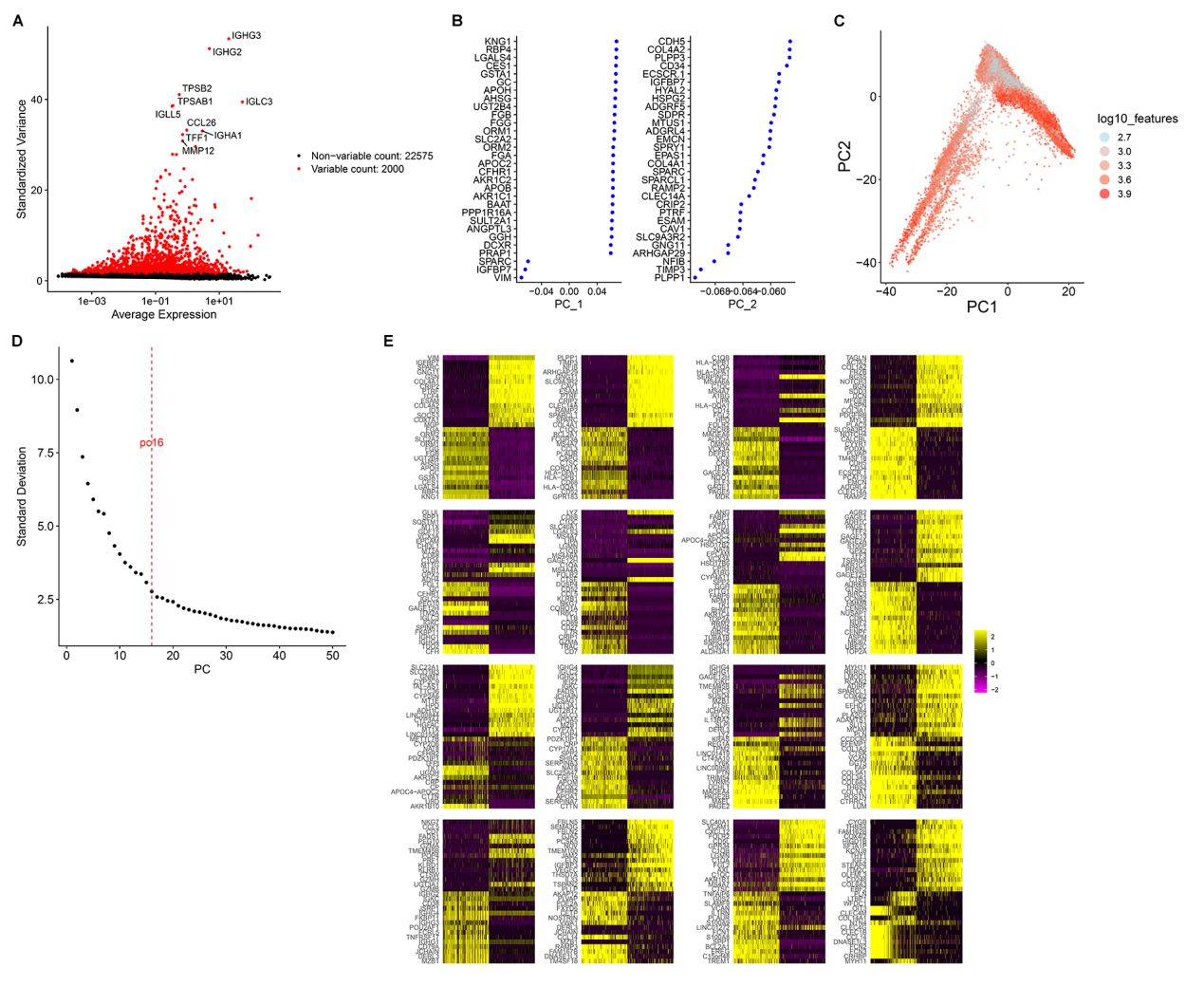
**

**Supplementary Figure S2. Data normalization of the scRNA-seq data** (A) Selection of the first 2000 highly variable genes in accordance with the standardized variance. (B) Exhibition of the first two PCs after linear dimension reduction analysis. (C) PCA plots of single cells. (D) Selection of the optimal number of PCs. (E) Heatmaps of the top 16 PCs.


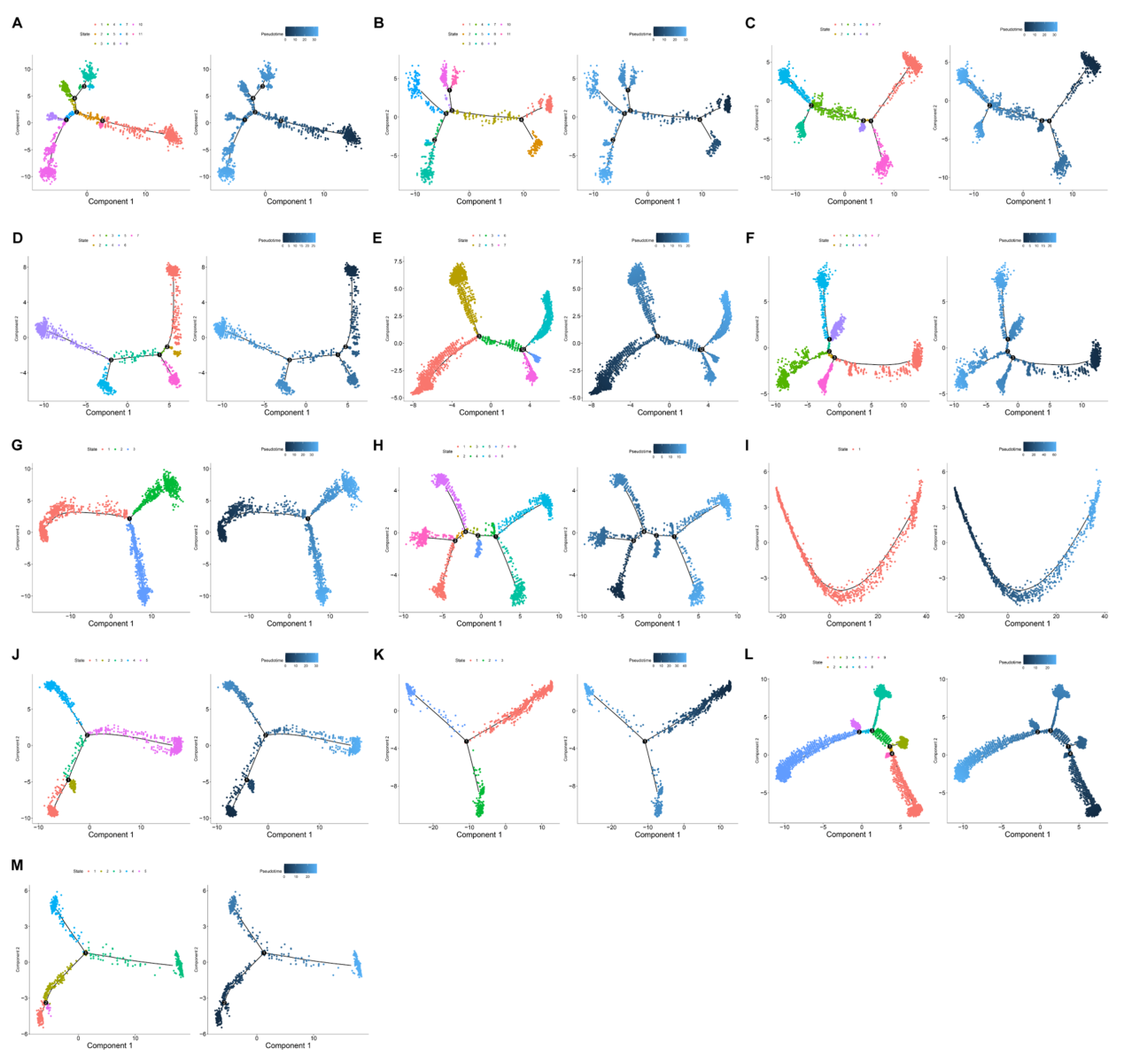


**Supplementary Figure S3. Trajectory analysis of each cell population**  (A) Cancer stem cells, (B) liver 2 stem cells, (C) progenitor cells, (D) mucosal cells, (E) CD^4+^ T cells, (F) CD8+ cytotoxic T cells, (G) Treg cells, (H) plasma cells, (I) naive B cells, (J) regulatory B cells, (K) natural killer 4 cells, (L) macrophages, and (M) M1 macrophages. Each dot denotes one cell.


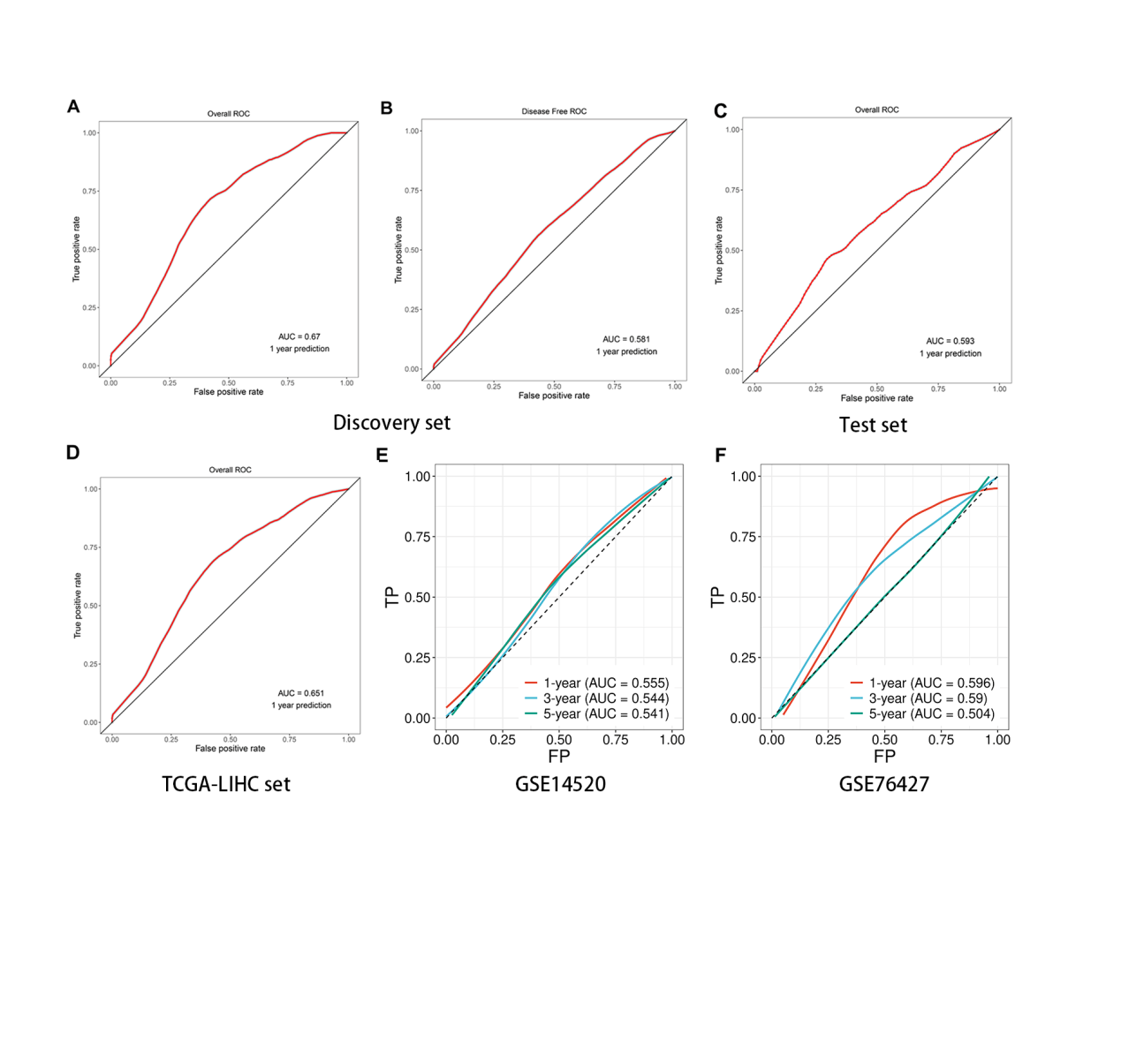


**Supplementary Figure S4.** ROC curves in the discovery set (A,B), test set (C), TCGA-LIHC set (D), GSE14520 (E) and GSE76427 (F)
