# Supplementary material for: Single-cell and bulk transcriptome analysis unveils a ligand-receptor-based signature for prognostication and reveals that TREM1 controls the malignant behaviors of hepatocellular carcinoma: Ligand-receptor prognostic signature and TREM1-driven HCC malignancy via single-cell/bulk analysis
Source: Acta Biochim Biophys Sin (Shanghai). 2025 Jun 23;57(11):1847–63. doi: 10.3724/abbs.2025059 (PMC12666666; doi:10.3724/abbs.2025059)
Supplement: 24895Supplementary_Table_1 [file 24895Supplementary_Table_1.docx]

**Supplementary Table S1. Clinical traits of HCC patients in the discover, test, and total sets**

| Parameters | TCGA-LIHC (*n* = 365) | Discovery set (*n* = 255) | Test set (*n* = 110) |
| --- | --- | --- | --- |
| Age | 59.65 ± 13.36 | 59.55 ± 13.5 | 59.86 ± 13.07 |
| Status |  |  |  |
| Alive | 235 (64.38) | 165 (64.71) | 70 (63.64) |
| Dead | 130 (35.62) | 90 (35.29) | 40 (36.36) |
| Sex |  |  |  |
| Male | 246 (67.4) | 172 (67.45) | 74 (67.27) |
| Female | 119 (32.6) | 83 (32.55) | 36 (32.73) |
| T stage |  |  |  |
| T0 | 1 (0.27) | 1 (0.39) | 0 (0) |
| T1 | 180 (49.32) | 116 (45.49) | 64 (58.18) |
| T2 | 91 (24.93) | 71 (27.84) | 20 (18.18) |
| T3 | 78 (21.37) | 56 (21.96) | 22 (20) |
| T4 | 13 (3.56) | 9 (3.53) | 4 (3.64) |
| TX | 1 (0.27) | 1 (0.39) | 0 (0) |
| Unknown | 1 (0.27) | 1 (0.39) | 0 (0) |
| N stage |  |  |  |
| N0 | 248 (67.95) | 172 (67.45) | 76 (69.09) |
| N1 | 4 (1.1) | 3 (1.18) | 1 (0.91) |
| NX | 112 (30.68) | 79 (30.98) | 33 (30) |
| Unknown | 1 (0.27) | 1 (0.39) | 0 (0) |
| M stage |  |  |  |
| M0 | 263 (72.05) | 186 (72.94) | 77 (70) |
| M1 | 3 (0.82) | 1 (0.39) | 2 (1.82) |
| MX | 99 (27.12) | 68 (26.67) | 31 (28.18) |
| Pathologic stage | |  |  |
| Stage I | 170 (46.58) | 109 (42.75) | 61 (55.45) |
| Stage II | 85 (23.29) | 67 (26.27) | 18 (16.36) |
| Stage III | 83 (22.74) | 62 (24.31) | 21 (19.09) |
| Stage IV | 5 (1.37) | 3 (1.18) | 2 (1.82) |
| Unknown | 22 (6.03) | 14 (5.49) | 8 (7.27) |

**Supplementary Table S2. Pseudotemporal ordering results of genes based on branched expression analysis modeling**

**Supplementary Table S3. Results of pseudotemporal function-based screening for differentially expressed genes**
